# Designing incentive mechanism in contract farming considering reciprocity preference

**DOI:** 10.1371/journal.pone.0269167

**Published:** 2022-06-06

**Authors:** Cuixia Wang, Yurong Liang

**Affiliations:** School of Information Management, Jiangxi University of Finance and Economics, Nanchang, China; University of Naples Federico II, ITALY

## Abstract

Contract farming is a growing practice in agricultural economy. A well-designed crop-planting and buyout contract, offered by an enterprise, to a fraction of contract farmers brings benefit to farmers as well as to the enterprise itself. However, in the process of contract fulfillment, farmers possess private information about the degree of effort on fulfilling the contract of themselves. Thus, the more informed farmers may not work hard in the process of planting crops. This opportunistic behavior of farmers caused by asymmetric information has seriously affected the sustainability of contract farming. An enterprise with reciprocity preference tends to make a contract both improves farmers’ welfare and brings enough profit to sustain its own operations, while a farmer with reciprocity will work hard in return for the enterprise’s reward. In this paper, we develop a non-profit index principal-agent model between enterprises and farmers, assuming both have reciprocity preference, to investigate how to design an incentive mechanism in contract farming. We begin our analysis by establishing a non-profit index evaluation system to evaluate farmers’ effort degree in contract fulfillment. Then we solve principal-agent problem with the assumption that farmers’ expected certainty income premium (ECIP) is constant. We find that in the perfect Bayesian equilibrium, farmers with higher degree of reciprocity preference require less ECIP, and will improve efforts to complete contract tasks, even actively sacrifice their own interests to repay extra rewards from enterprises. Furthermore, we explore our model to the scenario in which farmers’ ECIP is a function of enterprise payment difference (EPD). We find that the higher the degree of reciprocity preference of farmers, the greater the probability of enterprises to increase income. Numerical simulations are conducted to verify the validity of the conclusions. Our study shows that the reciprocity preference behavior of enterprises and farmers improves the fulfillment rate of contract farming, which contributes to the realization of the incentive mechanism of contract farming.

## 1. Introduction

Contract farming has been widely adopted in developed countries. For example, in the United States, more than 60% of large farms adopt the form of contract, and contract farming covers more than 40% of the output value of agricultural products every year [1, 2]. In recent years, it has become more popular in developing countries such as China, India, Brazil, and Turkey [3, 4]. Traditionally, farmers themselves make production decisions, and then sell agricultural commodities to the market. In contrast, contract farming is an agricultural production and operation mode, in which enterprises make a contract about agricultural products supply and sales with farmers after analyzing market demand [5]. Typically, farmers provide certain quantities of agricultural commodities at the specified quality standards and time, and enterprises commit to a specified pricing scheme and supply some inputs or technical support to farmers. Contract farming provides stable raw material supply and controllable quality of agricultural products for enterprises. Meanwhile, this kind of mode reduces the planting risk of farmers due to products unsalable and brings certain guarantee for the income of farmers. B shows that this form of private vertical coordination can be a key pillar of rural and agricultural development policy [6].

Though contract farming has several potential advantages, it encounters some challenges in practice. The typical problem faced by contract farming is the low contract fulfillment rate of farmers, which has caused great trouble for its development [7]. The past default cases of farmers in contract farming show that the low fulfillment rate of farmers is mainly reflected in its low degree of efforts, such as using hard-to-detect production hormones or falsely report production [8, 9]. Since agricultural products have the characteristics of experience and trust products, enterprises cannot directly detect the subtle differences in the quality of agricultural products. Further, farmers hide their true abilities, and enterprises cannot distinguish whether farmers have put in their full efforts. An information asymmetry exists between farmers and enterprises about the quality of agricultural products and the degree of farmers’ efforts. Farmers have private information. This can easily induce farmers to have opportunistic motives in contract farming, that is, to invest a lower degree of effort.

Take, as an example, the Starbucks company. The most important input of the company is unroasted coffee beans. In 2014, Starbucks bought more than half a billion pounds of these beans, contracting with more than 300,000 farmers worldwide in Latin America, Africa, and Asia [10]. Due to drought and crop diseases, coffee production in major coffee planting areas such as Brazil has dropped significantly in recent years. To provide enterprises with sufficient coffee beans, farmers reduced their degree of efforts driven by economic benefits, such as blending qualified coffee beans with lower-quality beans and failing to introduce new technologies in time [11]. Starbucks cannot test the coffee beans purchased from all over the world one by one due to the limitation of time and money, which ultimately affected the company’s revenue and caused great harm to the development of contract farming.

Low effort degree of farmers in fulfillment restricts the development of contract farming. To solve this problem, enterprises have set up mechanisms such as deferred payment mechanism and inspection mechanism to improve the degree of farmers’ effort [12]. Meanwhile, the government regulates the behavior of farmers by making laws and regulations or imposing fines or bonuses [13]. These measures have alleviated the phenomenon of the low degree of farmers’ effort, but they are all based on the assumption that farmers and enterprises are completely rational individuals, which contradict with the fact that both individuals in contract farming have certain reciprocity preference behavior characteristics in the reality. In order to fundamentally solve the low fulfillment rate of farmers with reciprocity preference in contract farming, it mainly establishes an incentive mechanism to improve farmers’ consciousness and efforts.

The theory of reciprocity preference was first put forward by behavioral economist Rabin in 1993. He pointed out that in modern economic society, people are not completely self-interested as Adam Smith said. To a certain extent, people will consider the interests of others and long for fairness, justice and equality [14]. In order to improve the degree of farmers’ efforts and promote the development of contract farming, many enterprises respond to the government’s policy for benefiting agriculture, set up priority channels of agricultural products and implement a series of measures with reciprocity preference. More examples are witnessed during recent years. In Chian, under the implementation of the government’s policy of benefiting farmers, JD.COM (China’s largest online retailer) in 2018, together with universities, established a national new agricultural talent training and export base to help cultivate new farmers [15]; In 2020, Alibaba announced "ten benefits to farmers" program to help farmers access to the market [16]. In Switzerland, SingularityNET, a decentralized artificial intelligence company, provided key agricultural information on the blockchain platform in 2019 to help farmers increase the annual production of crops [17].

In this paper, we develop a non-profit index principal-agent model, where both enterprises and farmers have the characteristic of reciprocity preference, to design an incentive mechanism in contract farming to improve the degree of farmers’ efforts in contract performance and the income of enterprises. We firstly build a non-profit index evaluation system to evaluate the degree of farmers’ effort. Compared with the traditional single profit indicator which probably increase the default risk of farmers, abundant non-profit evaluation indexes can more strictly restrict farmers’ behavior and scientifically reflect the quality of farmers’ fulfillment [18]. Then, combining the principal-agent theory and Rabin’s reciprocity preference theory, we build a principal-agent model considering reciprocity preference to study the incentive mechanism design of enterprises and farmers in contract farming. We analyze a simple model for the incentive mechanism with assuming the ECIP of farmers is constant and extend the model to assume farmers’ ECIP is a function of EPD. We address three main research questions:

(i) Can the reciprocity preference behavior of enterprises inspire farmers to improve their efforts?
(ii) Can the enterprise’s reciprocity preference for farmers ultimately increase its own income?
(iii) Facing farmers with different degree of reciprocity preference, what kind of incentive mechanism should enterprises design respectively?

In addition, we conduct a numerical study to verify the validity of the conclusions and some policy suggestions on how to improve the fulfillment rate of farmers are put forward to establish a more mature incentive mechanism of contract farming.

The rest of this paper is structured as follows. Section 2 reviews the past research relevant to our problem of study. Section 3 selects non-profit indexes to evaluate the degree of farmers’ effort and analyzes the ECIP of enterprises and farmers without considering reciprocity preferences. Section 4 constructs a non-profit index principal-agent framework with reciprocity preference to design an incentive mechanism to improve farmers’ effort degree in contract farming. We analyze a simple model for the incentive mechanism with assuming the ECIP of farmers is constant in section 4.1 and extend the model in section 4.2 to assume farmers’ ECIP is a function of EPD. Doing so has two benefits. First, it introduces the reader to the modeling framework used subsequently throughout the paper. Second, it establishes a baseline for the subsequent model. Section 5 conducts a numerical study to verify the operability of the conclusions. Section 6 concludes by discussing the main findings and ideas for future research.

## 2. Literature review

Our paper is closely related to the works on incentive-compatible mechanisms design considering reciprocity preference in contract farming. Due to the low fulfillment rate of farmers has caused great harm to the development of contract farming, a growing number of enterprises with reciprocity preference have set up incentive mechanisms to improve farmers’ effort degree. The design of incentive mechanism considering reciprocity preference in contract farming has been drawing great attention from researchers. We have conducted research on the above issue. To place our research in the appropriate context, the discussion is organized into three parts. The first part discusses the past literature regarding low implementation rate of farmers. The second part discusses the existing studies regarding the measures to improve the fulfillment rate of farmers. The third part discusses existing work regarding how the individual’s reciprocity preference influences its decisions.

### 2.1 Low implementation rate of farmers

Li [19] pointed out that the most prominent feature of contract farming could be the high default rate of farmers, and the source of default risk came from the incompleteness of the contract. Based on the survey data of 286 fruit farmers in Shandong Province, Guo [20] found that the number of farmers who did not meet the targets stipulated in the contract accounted for 46.1% of the total sample. Based on the effective questionnaire of 91 leading enterprises of agricultural industrialization, Wang et al. [21] made a theoretical discussion and empirical test on the order fulfillment efficiency and motivation of leading enterprises and farmers in contract farming. The results showed that the high default rate of farmers had become the bottleneck restricting the further development of China’s agricultural industrialization. Liu et al. [22] considered the multiple risks faced by agricultural production and investigated the contractual preference of farmers with heterogeneous risk attitude towards different types of risk sharing ability. They found that the contract fulfillment rate of risk-averse farmers was lower and this kind of farmers mainly focused on the degree of price risk sharing of the contract. Chen et al. [23] proposed that due to the uncertainty of the wholesale price, output, quality and other factors of agricultural products, farmers easily did not fulfill the contract.

The past research shows that due to factors such as climate change, uncertainty in agricultural production, and flawed contract design, the low fulfillment of famers is a common phenomenon. The low fulfillment rate of farmers has greatly restricted the development of contract farming, which has caused serious troubles to the growth of agricultural economy. Therefore, it is of great practical and scientific significance to study the farmers’ performance in contract farming.

### 2.2 Measures to improve the fulfillment rate of farmers

Some scholars proposed to improve the fulfillment rate of farmers by redesigning contracts. Ye et al. [24] designed a coordinated contract based on “revenue sharing + production cost sharing + margin contract”; Anderson et al. [25] designed a quantity discount contract for contract farming; Cao et al. [26] designed the wholesale price contract and the menu contract. Some scholars analyzed the factors of farmers’ default, and then proposed corresponding strategies to avoid farmers’ default [6, 23, 27]. Babich et al. [12] proposed that enterprises could set up mechanisms such as deferred payment mechanism and inspection mechanism to improve the degree of farmers’ effort. Levi et al. [13] put forward the government could regulate the behavior of farmers by making laws and regulations or imposing fines or bonuses.

The existing literature has mainly focused on the game relationship and principal-agent relationship between enterprises and farmers. Researchers combined with block-chain, big data and other cutting-edge knowledge and technology to study the characteristics of farmers’ default behavior, influencing factors of default, contract design defects and so on. Countermeasures and suggestions are put forward on how to improve the fulfillment rate of farmers from the perspectives of government, enterprises, and farmers.

### 2.3 Reciprocity preference

Most of the existing studies on contract farming assume that the individuals of contract farming are rational economic men whose decision-making criterion is to pursue the maximization of its own income. However, lots of empirical studies show that the individuals of contract farming have certain irrational fairness preference behavior characteristics when they participate in economic activities [28–32]. Therefore, it is more realistic to introduce non-self-interested psychological preferences such as reciprocity preference into the incentive mechanism of contract farming. Jiang et al. [33] found that service providers with social preference would charge the same price to different customers even if they had more information advantages. Du et al. [34] made a comparative study on the dynamic game of sharp and harmonious supply chain under the influence of reciprocity preference, and found that reciprocity preference made wholesale price contract unable to coordinate the sharp supply chain. Zhang [35] combined the reciprocity preference theory with the pricing decision analysis of closed-loop supply chain system, and found that the reciprocity preference behavior was conducive to the improvement of system revenue and channel efficiency. Ma et al. [36] studied the optimal contract design with fairness preference under the principal-agent framework, and the results showed that fair preference could motivate agents to make more efforts. Taking agricultural products electronic order pledge as an example, Xu [37] studied the incentive contract design between banks and B2B (Business-to-Business) platform by using principal-agent theory, and constructed a principal-agent model based on the reciprocity preference factors of B2B platform. He found that when considering the reciprocity preference, the stronger the preference behavior of B2B platform was, the higher the degree of effort would be, and the bank income only increased within the effective set.

In conclusion, the existing work on reciprocity preference behavior has been relatively mature, but it is still in its infancy to consider the reciprocal preference of enterprises and farmers in the field of contract farming.

Our paper differs from extant relevant publications in two aspects. First, we mainly study farmers’ low degree of efforts in the low fulfillment rate and select five non-profit indexes to evaluate the degree of farmers’ effort, which scientifically reflect the quality of farmers’ performance. Secondly, we consider both enterprises and farmers have the characteristic of reciprocity preference in contract farming. Combining the principal-agent theory and Rabin’s reciprocity preference theory, we build a principal-agent model considering reciprocity preference to study the incentive mechanism design of enterprises and farmers in contract farming.

## 3. Model assumption

We consider the contract farming mode which is composed of one enterprise and *n* farmers. The enterprise signs a contract with farmers about the production and sales of agricultural products. In the contract, the enterprise sets the production standards and assigns different tasks to farmers according to the characteristics of farmers and agricultural products. Accordingly, farmers organize and arrange the production of agricultural products. Apparently, there is a principal-agent relationship between the enterprise and *n* farmers. The enterprise is the principal and farmers are agents. According to the different risk characteristics of enterprises and farmers, we assume that the enterprise is the risk neutral and farmers are risk averse [38].

In the process of contract fulfillment, the enterprise often cannot fully know the real effort degree of the contracted farmers so that it’s necessary for the enterprise to evaluate the degree of farmers’ fulfillment efforts. We assume the enterprise establishes the valuation system consisting of *m* non-profit indexes to assess the effort degree of the tasks in contract that have been fulfilled by farmers, such as the agricultural product quality evaluation index, the agricultural product delivery index, farmers’ learning effort index and so on. To simplify the analysis, we assume that the larger the value of all indexes, the better the evaluation result of farmers will be.

Therefore, the fulfillment effort degree of farmer *i* is as follows:

$$E_{i}=\Sigma_{j=1}^{m}\left(\beta_{ij}q_{ij}+\varepsilon_{ij}\right),\;(i=1,2,\ldots ,n;j=1,2,\ldots ,m)$$ (1)

Where *β*_*ij*_ denotes the ability of farmer *i* to complete the index *j*, and the higher value of *β*_*ij*_ means that farmer *i* can complete the index more easily. *q*_*ij*_ represents the effort degree of farmer *i* on the input of the index *j*, and the greater the *q*_*ij*_ is, the more effort farmer *i* invests in the indicator *j* will be. For example, the index 1 of the non-profit evaluation system is the agricultural product quality evaluation index. Accordingly, *q*_11_ represents the effort degree of farmer 1 to achieve the quality standard, *β*_11_ is the ability of farmer 1 to complete the index 1, and *β*_11_*q*_11_ constitutes the effective effort level of farmer 1 on the index 1 fulfillment. *ε*_*ij*_ represents the market random variable that affects farmer *i* to complete the index *j*, which is subject to *N*(0, *σ*_*ij*_^2^) [39]. *β*_*ij*_, *ε*_*ij*_ is independent of each other.

According to the index effort degree of farmer *i*, the enterprise gives a certain reward $R_{i}=\gamma_{i}+\sum_{j=1}^{m}\alpha_{ij}(\beta_{ij}q_{ij}+\varepsilon_{ij})$. *γ*_*i*_ is the fixed pay provided by the enterprise to farmers and *α*_*ij*_ is the incentive coefficient for index fulfillment. The effort cost of the farmer *i* is assumed as $C_{i}=\frac{1}{2}a_{i}\sum_{j=1}^{m}{q_{ij}}^{2}$, *a*_*i*_ is the effort cost coefficient. This assumption has been commonly used in agricultural models [40, 41]. Hence, the net income of farmer *i* in contract farming is as

$$\Pi_{fi}=R_{i}-C_{i}=\gamma_{i}+\Sigma_{j=1}^{m}\alpha_{ij}\left(\beta_{ij}q_{ij}+\varepsilon_{ij}\right)-\frac{1}{2}a_{i}\Sigma_{j=1}^{m}q_{ij}{}^{2}$$ (2)

In the order task assigned to farmer *i*, the enterprise can obtain income $\Pi_{ei}=\sum_{j=1}^{m}\omega_{ij}(\beta_{ij}q_{ij}+\varepsilon_{ij})$. *ω*_*ij*_ is the contribution coefficient of the effective effort degree of the farmer *i* to complete the index *j* to the enterprise’s income. Therefore, the total income of enterprise in the whole contract farming is as

$$\Pi_{e}=\Sigma_{i=1}^{n}\left(\Pi_{ei}-R_{i}\right)=\Sigma_{i=1}^{n}\left[\Sigma_{j=1}^{m}(\omega_{ij}-\alpha_{ij}\right)\left(\beta_{ij}q_{ij}+\varepsilon_{ij}\right)-\gamma_{i}]$$ (3)

Since the enterprise is risk neutral, its determined equivalent income is equal to the expected value of its total income. Therefore, the equivalent income of the enterprise is written as

$$Z=E\left(\Pi_{e}\right)=\Sigma_{i=1}^{n}\left[\Sigma_{j=1}^{m}(\omega_{ij}-\alpha_{ij}\right)\beta_{ij}q_{ij}-\gamma_{i}]$$ (4)

The farmer *i* is the risk averse, and the risk cost it should bear is $C_{ri}=\frac{1}{2}b_{i}\sum_{j=1}^{m}{\alpha_{ij}}^{2}{\sigma_{ij}}^{2}$, *b*_*i*_ is the degree of risk aversion. Its equivalent income is written as

$$W_{i}=E\left(\Pi_{fi}\right)-C_{ri}=\gamma_{i}+\Sigma_{j=1}^{m}\alpha_{ij}\beta_{ij}q_{ij}-\frac{1}{2}a_{i}\Sigma_{j=1}^{m}q_{ij}{}^{2}\;-\frac{1}{2}b_{i}\Sigma_{j=1}^{m}\alpha_{ij}{}^{2}\sigma_{ij}{}^{2}$$ (5)

## 4. Incentive mechanism considering reciprocity preference

In this section, we consider that both enterprises and farmers have reciprocity preference behavior characteristics. Based on the existing research [37, 42], the reciprocity preference of the enterprise is to provide famers with extra rewards Δ*φ*_*i*_ which is also called the enterprise payment difference (EPD). Farmers with reciprocity preference will choose a higher degree of effort than the optimal effort degree in traditional model (without reciprocity preference) or even require less expected certainty income premium (ECIP) *θ*_*i*_ when they are motivated by the enterprise. Δ*q*_*ij*_ is the effort difference of the farmer *i* to complete the index *j* and $q_{ij}^{*}$ is the optimal effort degree of its traditional model. We suppose that *w*_*i*_ + *θ*_*i*_ represents the optimal equivalent income of farmer *i* in traditional mode. $w_{i}=\gamma_{i}+\sum_{j=1}^{m}\alpha_{ij}\beta_{ij}q_{ij}^{*}-\frac{1}{2}a_{i}\sum_{j=1}^{m}{q_{ij}^{*}}^{2}\;-\frac{1}{2}b_{i}\sum_{j=1}^{m}{\alpha_{ij}}^{2}{\sigma_{ij}}^{2}+\theta_{i}$, which is the retained income under the optimal effort degree of its traditional model, and *θ*_*i*_ is the ECIP of farmer *i*.

Therefore, considering reciprocity preference, the enterprise’s and the farmer’s equivalent income can be rewritten as

$$\tilde{Z}=E\left({\tilde{\Pi }}_{e}\right)-\Delta \varphi_{i}=\Sigma_{i=1}^{n}\left[\Sigma_{j=1}^{m}[\omega_{ij}\beta_{ij}(q_{ij}^{*}+\Delta q_{ij}\right)-\alpha_{ij}\beta_{ij}q_{ij}^{*}]-\gamma_{i}-\Delta \varphi_{i}]$$ (6)

$${\tilde{W}}_{i}=\gamma_{i}+\Sigma_{j=1}^{m}\alpha_{ij}\beta_{ij}q_{ij}^{*}+\Delta \varphi_{i}-\frac{1}{2}a_{i}\Sigma_{j=1}^{m}q_{ij}^{*}{}^{2}-\frac{1}{2}a_{i}\Sigma_{j=1}^{m}\Delta q_{ij}{}^{2}-\frac{1}{2}b_{i}\Sigma_{j=1}^{m}\alpha_{ij}{}^{2}\sigma_{ij}{}^{2}$$ (7)

Hereafter, we use the symbol “~” to denote the reciprocity preference. $\tilde{Z}$ and ${\tilde{W}}_{i}$ indicate the enterprise’s and farmer’s deterministic equivalent income when they have reciprocal preference, respectively.

In order to analyze the influence of farmers’ reciprocity preference on the results of the model, we study the incentive mechanism design of contract farming from two aspects: whether farmers improve their efforts with sacrificing their own interests to repay the "Friendliness" of the enterprise. That is, farmers’ ECIP is a constant and farmers’ ECIP is a function of the EPD.

### 4.1 Farmers’ ECIP is constant

In section 4.1, we analyze the design of incentive mechanism in contract farming when the certainty income premium expected by farmers is a constant. Farmers with reciprocity preference repay the enterprise’s extra reward Δ*φ*_*i*_ by improving the degree of their efforts to complete order tasks.

In the environment of asymmetric information of agricultural products, the incentive mechanism set up by the enterprise should not only meet the incentive constraints of farmers to actively complete orders, but also meet the expectation of optimal return obtained by reciprocity preference behavior under the existence of moral hazard. Based on the principal-agent theory and the above analysis hypothesis, the enterprise can maximize its income under the condition that farmers meet incentive constraint and participation constraint. Therefore, the incentive mechanism model of the enterprise to farmers is constructed as follows:

$$\text{Max}\;\tilde{Z}=E\left({\tilde{\Pi }}_{e}\right)-\Delta \varphi_{i}$$ (8a)

$$\text{s}.\text{t}.\;IC:\;\;{\tilde{W}}_{i}\geq w_{i}+\theta_{i}$$ (8b)

$$IR:\;q_{ij}\in argmax\;\;{\tilde{W}}_{i}$$ (8c)

Where the Eq (8b) is incentive compatibility constraint, and the Eq (8c) means the incentive rationality. *w*_*i*_ + *θ*_*i*_ represents the optimal equivalent income of farmer *i*, *w*_*i*_ is the retained income under the optimal effort degree of its traditional model and *θ*_*i*_ is the ECIP of farmer *i*.

By solving the above equations, we can get the following propositions.

#### Proposition 1

When the extra reward Δ*φ*_*i*_ is higher than *θ*_*i*_, farmer *i* with reciprocity preference will increase the degree of fulfillment effort, that is, Δ*q*_*ij*_ ≥ 0.

Proof. We can expand the above Eqs (8a)–(8c) as:

$$\text{Max}\;\tilde{Z}=\Sigma_{i=1}^{n}\left[\Sigma_{j=1}^{m}[\omega_{ij}\beta_{ij}(q_{ij}^{*}+\Delta q_{ij}\right)-\alpha_{ij}\beta_{ij}q_{ij}^{*}]-\gamma_{i}-\Delta \varphi_{i}]$$ (9a)

$$\text{s}.\text{t}.\;\gamma_{i}+\Sigma_{j=1}^{m}\alpha_{ij}\beta_{ij}q_{ij}^{*}+\Delta \varphi_{i}-\frac{1}{2}a_{i}\Sigma_{j=1}^{m}q_{ij}^{*}{}^{2}-\frac{1}{2}a_{i}\Sigma_{j=1}^{m}\Delta q_{ij}{}^{2}$$

$$-\frac{1}{2}b_{i}\Sigma_{j=1}^{m}\alpha_{ij}{}^{2}\sigma_{ij}{}^{2}\geq \gamma_{i}+\Sigma_{j=1}^{m}\alpha_{ij}\beta_{ij}q_{ij}^{*}$$

$$-\frac{1}{2}a_{i}\Sigma_{j=1}^{m}q_{ij}^{*}{}^{2}\;-\frac{1}{2}b_{i}\Sigma_{j=1}^{m}\alpha_{ij}{}^{2}\sigma_{ij}{}^{2}+\theta_{i}$$ (9b)

$$q_{ij}\in argmax\;\;{\tilde{W}}_{i}=\gamma_{i}+\Sigma_{j=1}^{m}\alpha_{ij}\beta_{ij}q_{ij}^{*}+\Delta \varphi_{i}-\frac{1}{2}a_{i}\Sigma_{j=1}^{m}q_{ij}^{*}{}^{2}$$

$$-\frac{1}{2}a_{i}\Sigma_{j=1}^{m}\Delta q_{ij}{}^{2}-\frac{1}{2}b_{i}\Sigma_{j=1}^{m}\alpha_{ij}{}^{2}\sigma_{ij}{}^{2}$$ (9c)

Replace $\sum_{j=1}^{m}{{\Delta q}_{ij}}^{2}$ with *m*Δ*q*_*i*_^2^ and combine Eqs (9a)–(9c), we can obtain: ${\Delta q}_{i}=\sqrt{\frac{2(\Delta \varphi_{i}-\theta_{i})}{ma_{i}}}$. It can be seen from the above equation: when Δ*φ*_*i*_ ≥ *θ*_*i*_, Δ*q*_*i*_ ≥ 0. That is, when the EDP Δ*φ*_*i*_ is higher than certainty income premium expected by farmer *i*, farmer *i* will put extra efforts Δ*q*_*i*_.

Proposition 1 proves the reciprocity preference behavior of the enterprise, that is, providing extra reward, can effectively encourage farmers to improve their efforts. The reciprocity preference behavior of the enterprise is meaningful and practical for designing incentive mechanism in contract farming.

For example, JD.COM cooperates with universities to cultivate new farmers. After learning new knowledge and technologies, farmers improve the output of agricultural products to promote the development of contract farming.

We then analyze the effect of the reciprocity preference degree of farmer *i* on its expected certainty income premium. Partial derivative of Δ*q*_*i*_ to *θ*_*i*_, we can get $\frac{\partial {\Delta q}_{i}}{\partial \theta_{i}}=-\frac{1}{\sqrt{2ma_{i}({\Delta \varphi }_{i}-\theta_{i})}}<0$. We have the following proposition.

#### Proposition 2

The higher the degree of reciprocity preference of farmer *i* has, the smaller *θ*_*i*_ will be and the more extra efforts farmer *i* will put in.

The above proposition shows that *θ*_*i*_ can be used to describe the degree of farmers’ reciprocity preference. The lower *θ*_*i*_ is, the higher the degree of reciprocity preference of farmer *i* will be. On the condition that the enterprise provides the same extra reward, farmers with higher degree of reciprocity preference have stronger dedication spirit. While increasing the effort degree, such farmers will even take the initiative to sacrifice their own interests, that is, to reduce the expected certainty income premium, and actively cooperate with the requirements of the enterprise.

Next, we investigate the impact of the reciprocity preference of farmer *i* on the income of the enterprise. The results are summarized in proposition 3.

#### Proposition 3

When the ECIP of farmer *i* is lower than the threshold ${\hat{\theta }}_{i}$, its reciprocal behavior will increase the enterprise’s income; when the ECIP of farmer *i* is higher than the threshold ${\hat{\theta }}_{i}$, the enterprise’s income depends on the ability of farmer *i* to complete the indexes *β*_*i*_, the index contribution coefficient *ω*_*i*_ and the effort cost coefficient *a*_*i*_.

Proof. *mω*_*i*_*β*_*i*_, *mq*_*i*_ and *mα*_*i*_ represents $\sum_{j=1}^{m}\omega_{ij}\beta_{ij},\;\sum_{j=1}^{m}q_{ij}^{*}$ and $\sum_{j=1}^{m}\alpha_{ij}$, respectively. When farmer *i* complies with the reciprocity preference, the income of the enterprise is as follows:

$$\tilde{Z}=\Sigma_{i=1}^{n}\left[m\omega_{i}\beta_{i}\left(q_{i}+\sqrt{\frac{2\left(\Delta \varphi_{i}-\theta_{i}\right)}{ma_{i}}}-\right)-m\alpha_{i}\beta_{i}q_{i}-\gamma_{i}-\Delta \varphi_{i}\right]$$ (10)

The enterprise can maximize its profits by obtaining the best payment difference. By calculating the partial derivative of the Eq (10) to Δ*φ*_*i*_, we can get the following results: $\frac{\partial \tilde{Z}}{\partial {\Delta \varphi }_{i}}=\sum_{i=1}^{n}\left[\sqrt{\frac{m{\omega_{i}}^{2}{\beta_{i}}^{2}}{2a_{i}({\Delta \varphi }_{i}-\theta_{i})}}-1\right]$. Let $\frac{\partial \tilde{Z}}{\partial {\Delta \varphi }_{i}}=0$, we get ${\Delta \varphi }_{i}=\frac{m{\omega_{i}}^{2}{\beta_{i}}^{2}}{2a_{i}}+\theta_{i}$. The calculation results are brought into the Eq (10), we get

$$\tilde{Z}=\Sigma_{i=1}^{n}[m\beta_{i}q_{i}\left(\omega_{i}-\alpha_{i}\right)+\frac{m\omega_{i}{}^{2}q_{i}{}^{2}}{2}-\gamma_{i}-\theta_{i}]$$ (11)

When farmer *i* is rational, the income of the enterprise is as follows:

$$Z=\Sigma_{i=1}^{n}\left[m\beta_{i}\left(\omega_{i}-\alpha_{i}\right)q_{i}-\gamma_{i}\right]$$ (12)

By comparing the Eq (11) with the Eq (12), we can get the following results:

$$\tilde{Z}-Z=\Sigma_{i=1}^{n}\left[\frac{m\omega_{i}{}^{2}\beta_{i}{}^{2}}{2a_{i}}-\theta_{i}\right]$$ (13)

When the condition $\frac{m{\omega_{i}}^{2}{\beta_{i}}^{2}}{2a_{i}}\geq \theta_{i}$ holds, we can get $\tilde{Z}-Z>0$ and the threshold ${\hat{\theta }}_{i}=\frac{m{\omega_{i}}^{2}{\beta_{i}}^{2}}{2a_{i}}$.

Proposition 3 illustrates that when farmers have high reciprocal preference behavior characteristics, the reciprocity preference behavior of enterprises can ultimately benefit enterprises themselves. This explains why in the real economy and society, enterprises are willing to provide some convenient behaviors for contract farmers.

Hema, Alibaba’s Freshippo Store, promoted the development of agricultural information technology by combining with the Internet. From agricultural materials, planting technology to a complete set of supply chain solutions, it has helped farmers reduce planting costs, improve production, and ensure the quality and quantity of contract farming. Finally, the reciprocity preference behavior of Hema increased its income.

### 4.2 Farmers’ ECIP and EPD are functional relations

In this section, we analyze the design of incentive mechanism in contract farming when the certainty income premium expected by farmers and enterprise payment difference are functional relations.

According to Proposition 2, farmers with higher reciprocity preference will require less certainty income premium *θ*_*i*_. When they receive more "goodwill" from enterprises, as feedback, they will improve the degree of efforts to complete the order task, and even take the initiative to sacrifice their own interests to repay the "goodwill" of enterprises. It can be concluded that the certainty income premium of farmer *i θ*_*i*_ will change with the change of the variable Δ*φ*_*i*_. There is a reciprocity function between the enterprise and farmer, which is consistent with the research of Soble [43]. Differently, we suppose the functional relationship between them is *θ*_*i*_ = *y*(Δ*φ*_*i*_), *y*′(Δ*φ*_*i*_) ≤ 0. Under the above premise, the equivalent income of the enterprise is as follows

$$\tilde{Z}=\Sigma_{i=1}^{n}\left[m\omega_{i}\beta_{i}\left(q_{i}+\sqrt{\frac{2\left(\Delta \varphi_{i}-y\left(\Delta \varphi_{i}\right)\right)}{ma_{i}}}\right)-m\alpha_{i}\beta_{i}q_{i}-\gamma_{i}-\Delta \varphi_{i}\right]$$ (14)

Using the Eq (14) replace the Eq (9a), we resolve the Eqs (9a)–(9c) and obtain the following proposition.

#### Proposition 4

Under the condition that the enterprise provides the same additional remuneration Δ*φ*, the higher the degree of reciprocity preference of farmers is, the smaller *y*(Δ*φ*_*i*_) will be, and the greater the possibility of increasing the enterprise’s income will be.

Proof. The Eq (14) is used to calculate the partial derivative of Δ*φ*_*i*_, we get $\frac{\partial \tilde{Z}}{\partial {\Delta \varphi }_{i}}=\sum_{i=1}^{n}\left[\frac{\omega_{i}\beta_{i}}{a_{i}}\frac{\left(1-y^{\prime }\left({\Delta \varphi }_{i}\right)\right)}{a_{i}\sqrt{\frac{2({\Delta \varphi }_{i}-y\left({\Delta \varphi }_{i}\right))}{ma_{i}}}}-1\right]$. Let the equation $\frac{\partial \tilde{Z}}{\partial {\Delta \varphi }_{i}}=0$ hold. By solving this equation, we can get ${\Delta \varphi }_{i}=\frac{{m{\omega_{i}}^{2}{\beta_{i}}^{2}(1-y^{\prime }\left({\Delta \varphi }_{i}\right))}^{2}}{2a_{i}}+y\left({\Delta \varphi }_{i}\right)$. At this time, the equivalent income of the enterprise is as

$$\Delta \tilde{Z}=\tilde{Z}-Z=\Sigma_{i=1}^{n}\left[\frac{m\omega_{i}{}^{2}\beta_{i}{}^{2}(1-y^{\prime }\left(\Delta \varphi_{i}\right)\left(\frac{1}{2}+y^{\prime }\left(\Delta \varphi_{i}\right)\right)}{a_{i}}-y\left(\Delta \varphi_{i}\right)\right]$$ (15)

The higher the degree of reciprocity preference of farmers is, the smaller the value of *y*(Δ*φ*_*i*_) will be. When the value of *y*(Δ*φ*_*i*_) is extremely small, the constraint $\frac{m{\omega_{i}}^{2}{\beta_{i}}^{2}(1-y^{\prime }\left({\Delta \varphi }_{i}\right)(\frac{1}{2}+y^{\prime }\left({\Delta \varphi }_{i}\right))}{a_{i}}-y\left({\Delta \varphi }_{i}\right)\geq 0$ is satisfied. Thus, with the increase of farmers’ efforts, the probability of enterprises getting extra income will increase.

We further analyze the impact of the degree of farmers’ reciprocity preference on the added value of enterprise income, and the following proposition can be drawn.

#### Proposition 5

Assuming that other variables are the same, under the condition that the EPD Δ*φ*_*i*_ is same, the more efforts the farmers with higher reciprocity preference put in, the higher the increased income of the enterprise $\Delta \tilde{Z}$ will be.

Proof. Different farmers have different degrees of reciprocity preference, that is, *θ*_1_ ≠ *θ*_2_. If the degree of reciprocity preference of farmer 1 is higher than that of farmer 2, in the case of the same additional payment by the enterprise, we can get *y*_1_(Δ*φ*_*i*_) ≤ *y*_2_(Δ*φ*_*i*_), Δ*q*_1_ ≥ Δ*q*_2_. According to the calculation, it can be concluded that: ${\Delta \tilde{Z}}_{1}\geq {\Delta \tilde{Z}}_{2}$.

Proposition 5 describes how much is the increase in enterprises’ income, which is related to farmers’ degree of reciprocity preference. Farmers with higher degree of reciprocity preference will make more efforts. Therefore, even if enterprises provide a lower extra reward, they will actively cooperate with the requirements of enterprises and put into the best effort degree. Therefore, in contract farming, enterprises could provide lower additional remuneration to farmers with higher degree of reciprocity preference to save costs.

Amazon.com, world’s largest e-commerce company, created the “Amazon Fresh” in which providing free cold chain logistics for farmers. In return, farmers with reciprocity preference provided fresh agricultural products. High quality agricultural products create more commercial space for Amazon.com.

## 5. Numerical study

In this section, we conduct a numerical study to investigate how the reciprocal preference behavior of the enterprise affects farmers’ efforts and the income of itself.

In order to more intuitively highlight the operability of the results of the paper, we assume a set of data to illustrate. To simplify, we consider a contract farming consisting of one enterprise and two farmers denoted by *i* = 1, 2. Two indexes, denoted by *j* = 1, 2, are used to evaluate the degree of farmers’ effort.

Regarding parameter setting, we used the parameter calibration method of the neoclassical school and referred to the parameter assignment idea of the existing research [14, 42, 44, 45]. We assume the ability of the same farmer to complete the two indexes and the effort degree of the same farmer on the input of the two indexes are the same, namely *β*_11_ = *β*_12_ = 5, *β*_21_ = *β*_22_ = 10, *q*_11_ = *q*_12_ = 10, *q*_21_ = *q*_22_ = 12. Since the value of contribution coefficient of tasks is in the range [0, 1], we assume the contribution coefficient of the two tasks to the order revenue is the same, that is, *ω*_1*j*_ = *ω*_2*j*_ = 0.5. Similarly, the coefficient of effort cost and the degree of risk aversion of the two farmers are the same, i.e., *a*_1_ = *a*_2_ = 0.2, *b*_1_ = *b*_2_ = 0.3. Market random factors have the same effect on task completion, that is, *ε*_*ij*_ is subject to *N*(0,1000). The incentive coefficient of task completion is the same, i.e., $\alpha_{11}=\alpha_{12}=\frac{\omega_{1j}{\beta_{1j}}^{2}}{{\beta_{1j}}^{2}+a_{1}b_{1}{\delta_{1j}}^{2}}=0.15,\;\alpha_{21}=\alpha_{22}=\frac{\omega_{2j}{\beta_{2j}}^{2}}{{\beta_{2j}}^{2}+a_{2}b_{2}{\delta_{2j}}^{2}}=0.31$.

When certainty income premium expected by farmers is constant, the added income of the enterprise is as $\Delta \tilde{Z}=\sum_{i=1}^{n}\left[\frac{m{\omega_{i}}^{2}{\beta_{i}}^{2}}{2a_{i}}-\theta_{i}\right]$. We assume that only farmer 1 exists in contract farming, and the added income of the enterprise who have implemented reciprocity preference is $\Delta \tilde{Z}=\frac{2{\omega_{1}}^{2}{\beta_{1}}^{2}}{2a_{1}}-0.2$.

In order to characterize intuitively the impacts of variables on the enterprise’s added income $\Delta \tilde{Z}$, we fix the other variables and study the impact of a single variable on the increase in enterprise’s income. The numerical simulation results are shown in the following figures.

**Fig 1:**
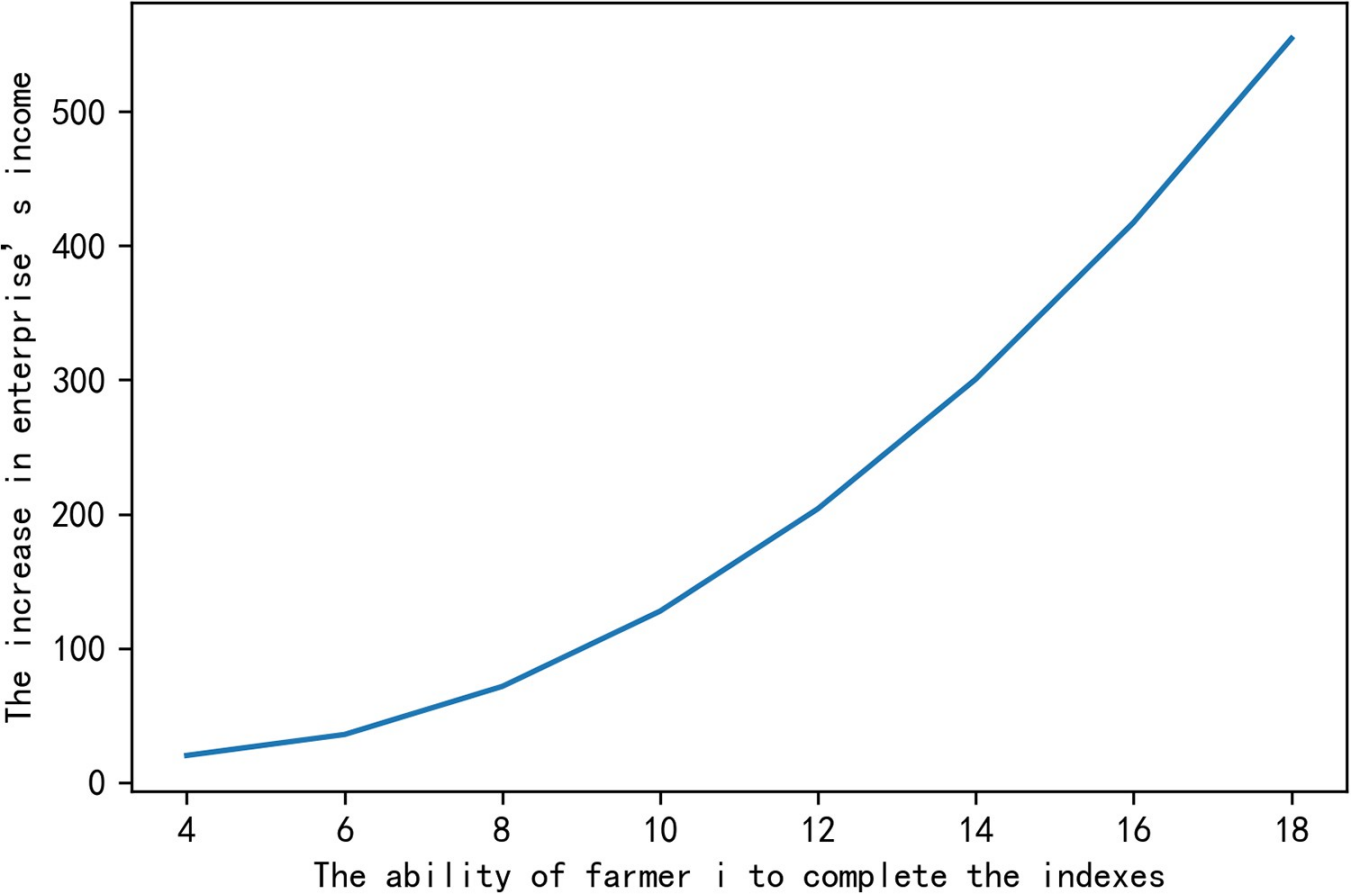
The impact of farmers’ ability to complete indexes on the increase in enterprise’s income.

**Fig 2:**
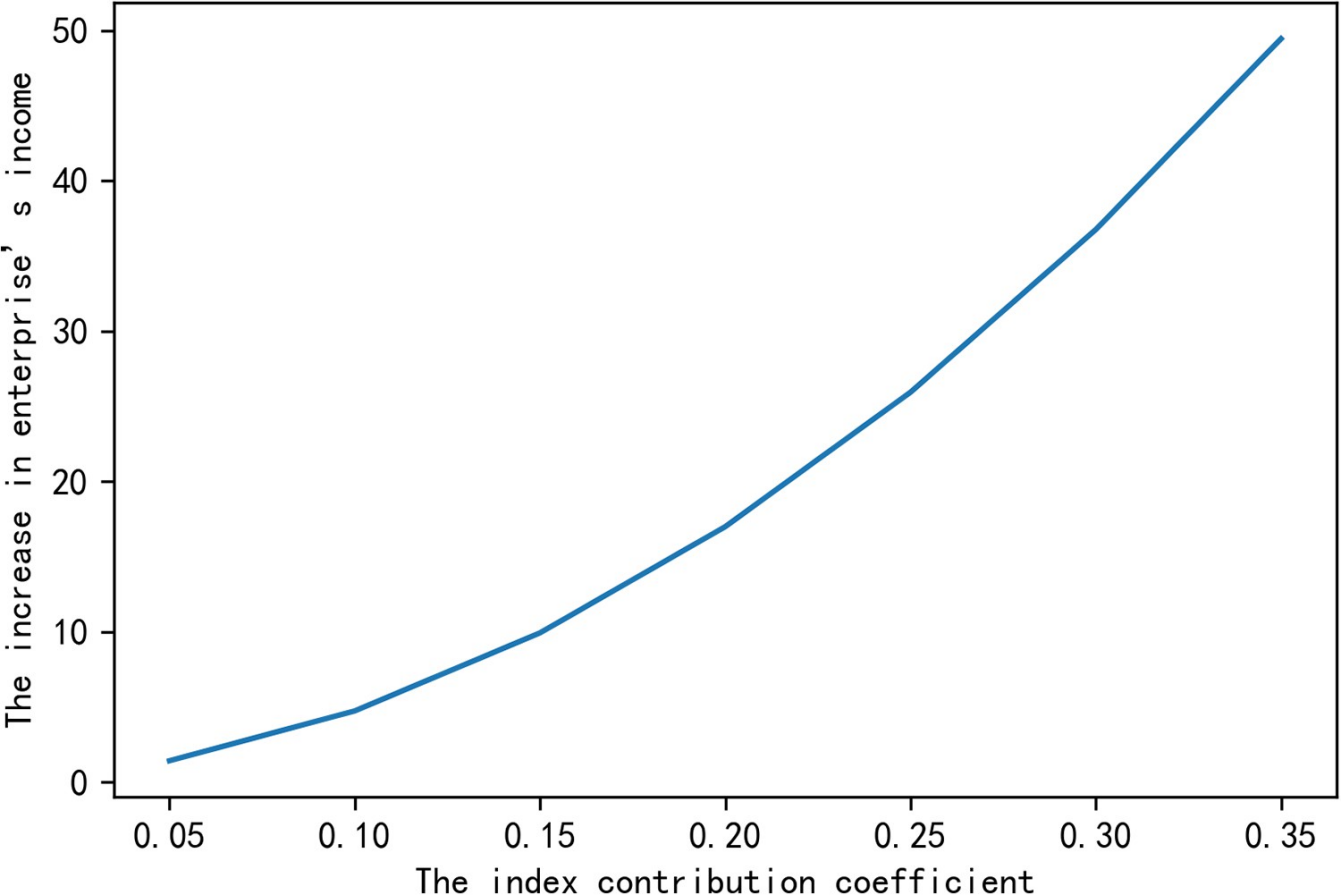
The impact of the index contribution coefficient on the increase in enterprise’s income.

**Fig 3:**
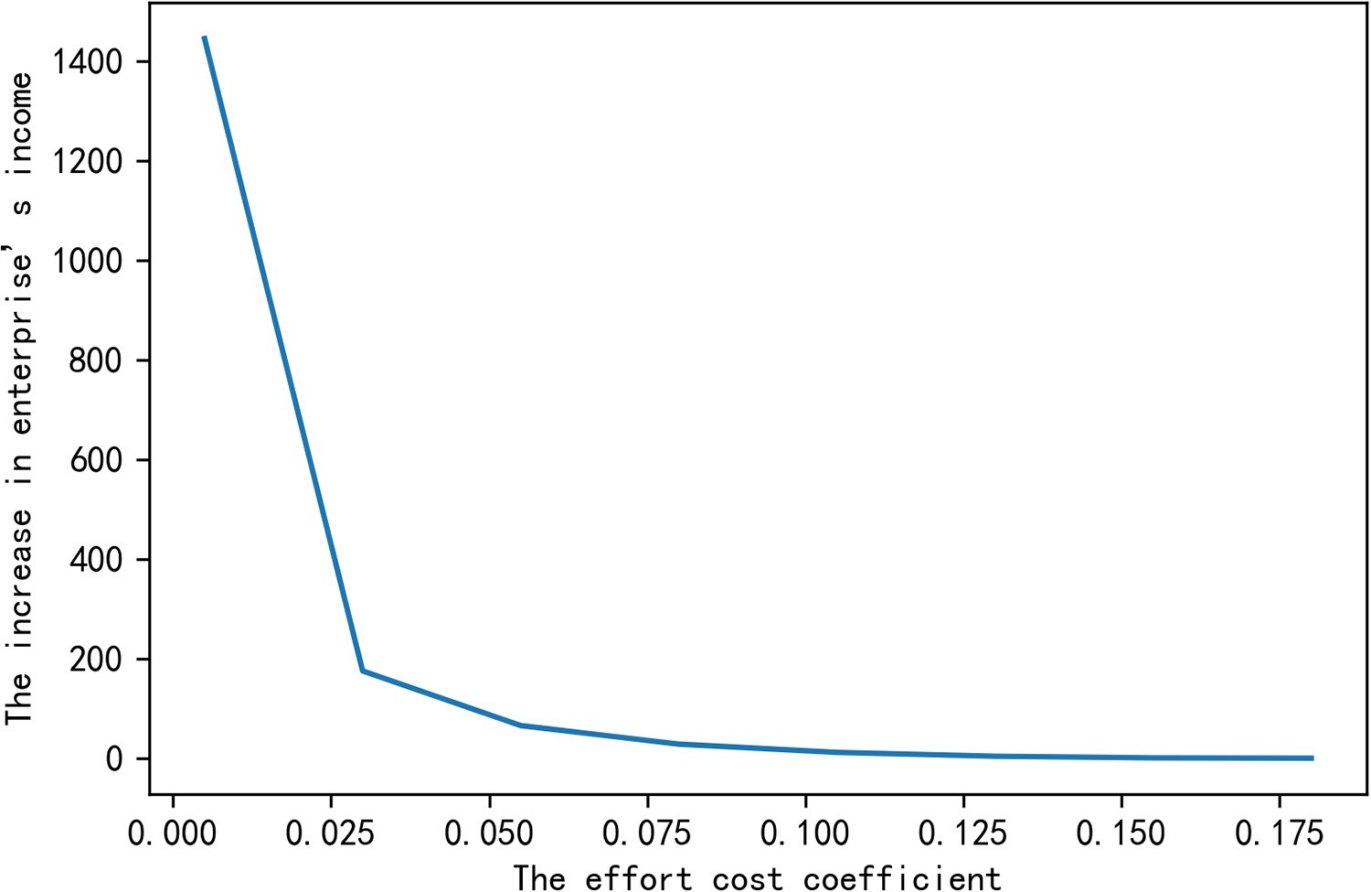
The impact of the effort cost coefficient on the increase in enterprise’s income.

From Figs 1–3, we get that when certainty income premium expected by farmers is constant, the increase of the enterprise’s income depends on the ability of farmer *i* to complete the indexes *β*_*i*_, the index contribution coefficient *ω*_*i*_ and the effort cost coefficient *a*_*i*_. Fig 1 shows that the greater the ability of farmers to complete indexes is, the greater the probability of the increase in enterprise’s income will be. Fig 2 proves that the increase in enterprise’s income $\Delta \tilde{Z}$ is proportional to the index contribution coefficient *ω*_*i*_, that is, the greater the index contribution coefficient is, the higher the increase in enterprise’s income will be. Fig 3 shows that the effort cost of farmers is inversely proportional to the increase in enterprise’s income. These conclusions are consistent with reality, which shows that the conclusions we have reached is practical.

**Fig 4:**
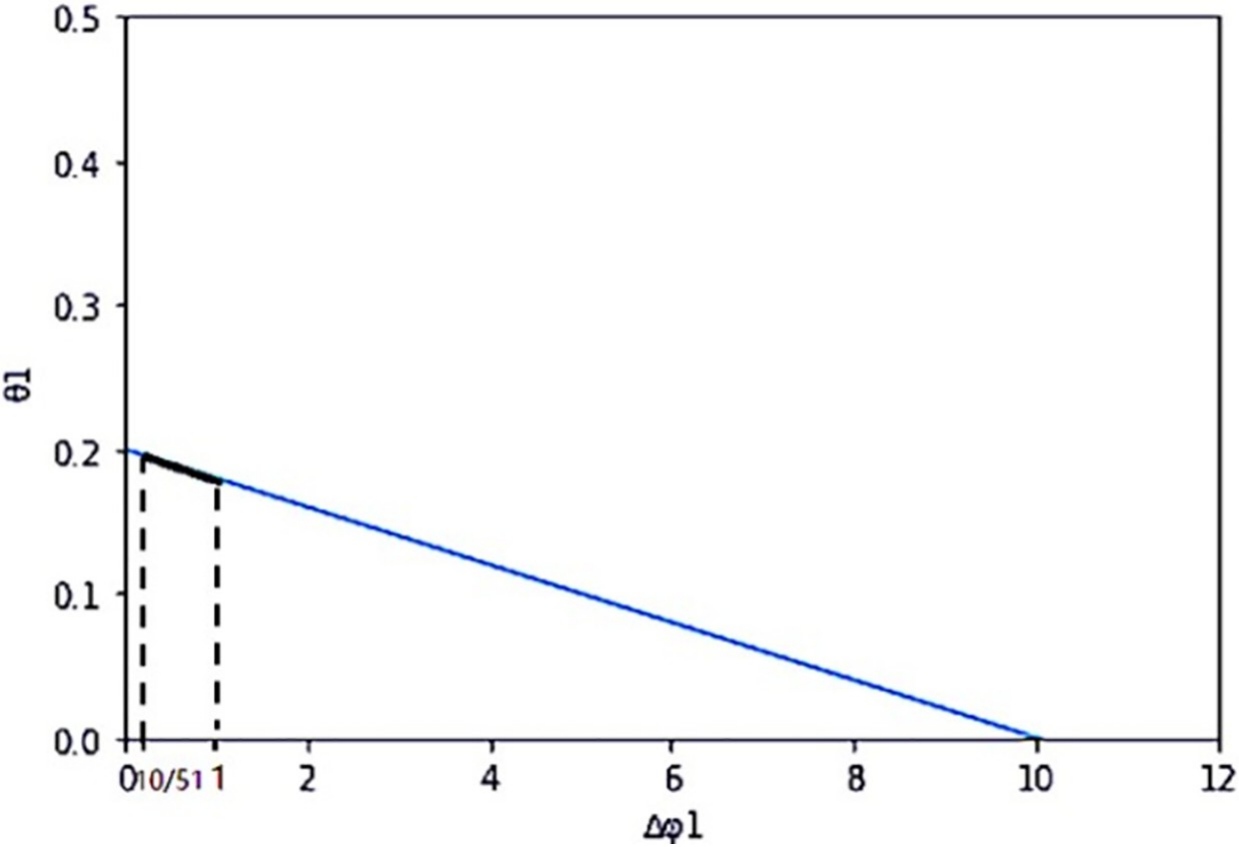
The functional relationship between *θ*_1_ and Δ*φ*_1_.

**Fig 5:**
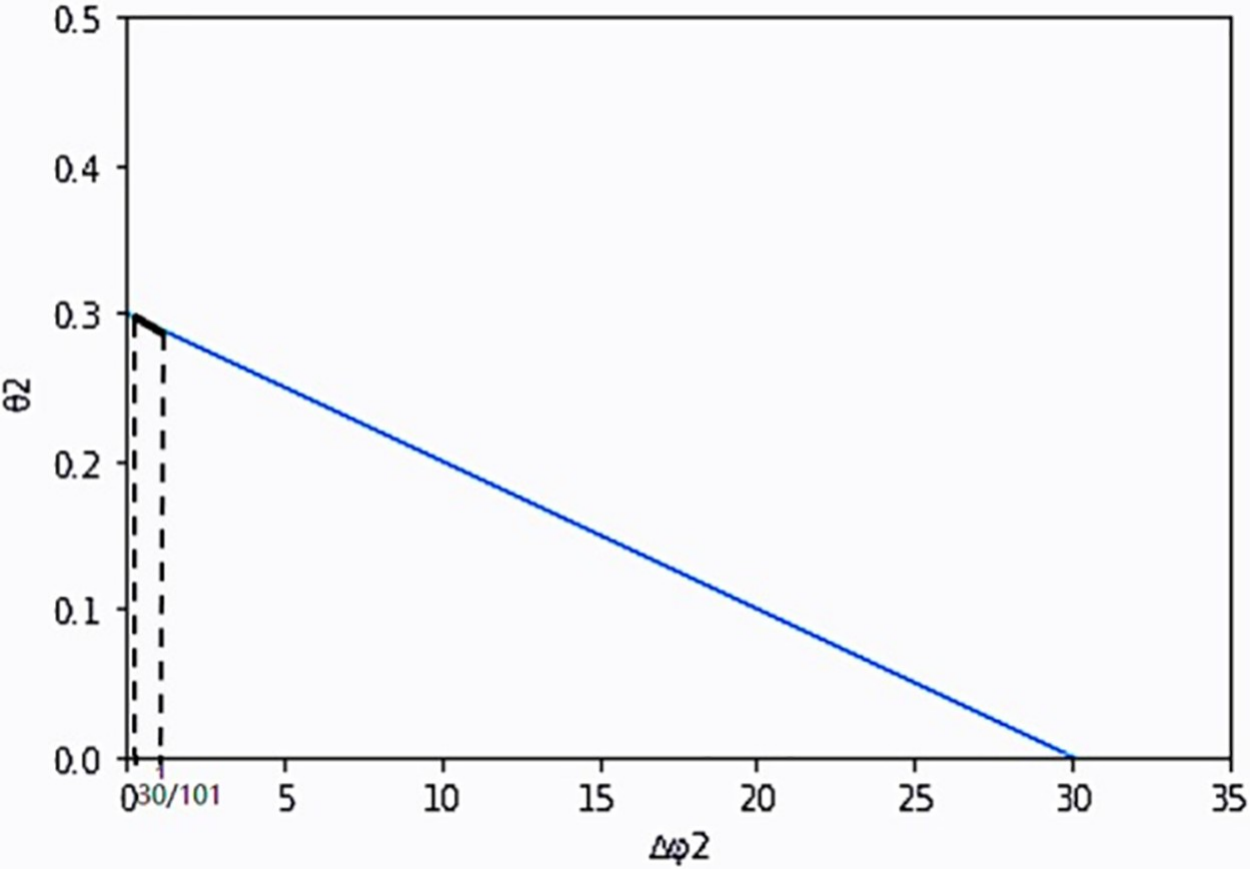
The functional relationship between *θ*_2_ and Δ*φ*_2_.

When the certainty income premium expected by farmers and enterprise payment difference are functional relations, we suppose the function expression between the extra payment difference of enterprises Δ*φ*_*i*_ and the certainty income premium expected by farmers *θ*_*i*_ is as follows: *θ*_1_ = *y*_1_ (Δ*φ*_1_) = −0.02Δ*φ*_1_ + 0.2, *θ*_2_ = *y*_2_ (Δ*φ*_2_) = −0.01Δ*φ*_2_ + 0.3. It can be concluded that: *y*′(Δ*φ*_1_) = −0.02, *y*′(Δ*φ*_2_) = −0.01. If the above assumed values are brought into Eq (14), we can get: $\tilde{Z}=Z+3.89-y_{1}\left({\Delta \varphi }_{1}\right)-y_{2}\left({\Delta \varphi }_{2}\right)$, the added income of the enterprise is $\Delta \tilde{Z}=\tilde{Z}-Z=3.89-y_{1}\left({\Delta \varphi }_{1}\right)-y_{2}\left({\Delta \varphi }_{2}\right)$. In order to make the results have practical significance, it is necessary to meet the conditions: Δ*φ*_*i*_ ≥ 0, *θ*_*i*_ ≥ 0, *θ*_*i*_ ≤ Δ*φ*_*i*_. Therefore, the effective range of payment difference between the two farmers can be obtained as follows: ${\Delta \varphi }_{1}\in (\frac{10}{51},\;1)$ and ${\Delta \varphi }_{2}\in (\frac{30}{101},\;1)$. In the range of valid set, the corresponding *y*_*i*_(Δ*φ*_*i*_) value is *y*_1_(Δ*φ*_1_) ∈ (0.18, 0.20), *y*_2_(Δ*φ*_2_) ∈ (0.29, 0.30). The changes of the two variables are shown in Figs 4 and 5, and the effective set is the thick line part in the graph.

**Fig 6:**
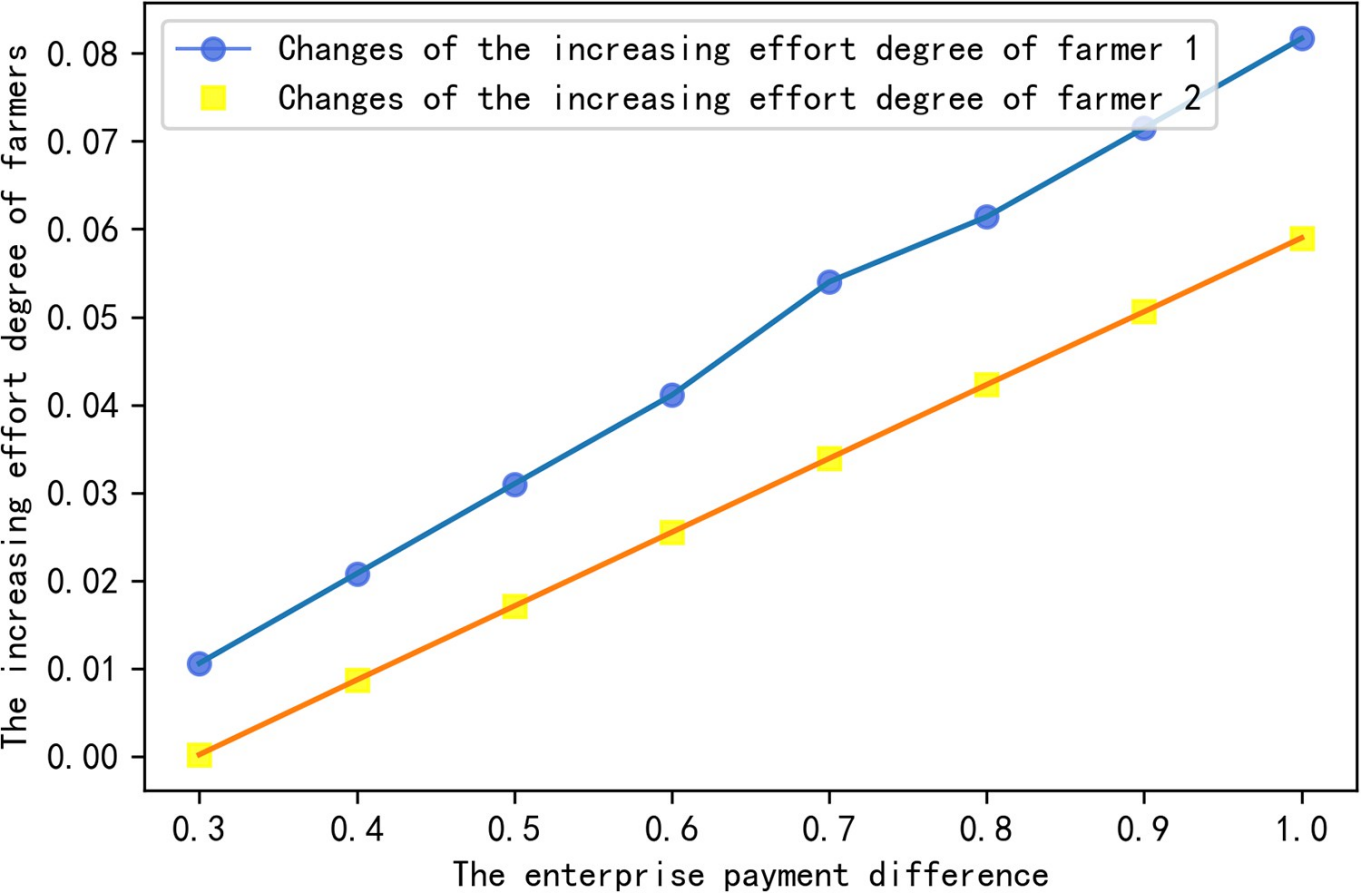
The relationship between farmers’ effort and EPD.

**Fig 7:**
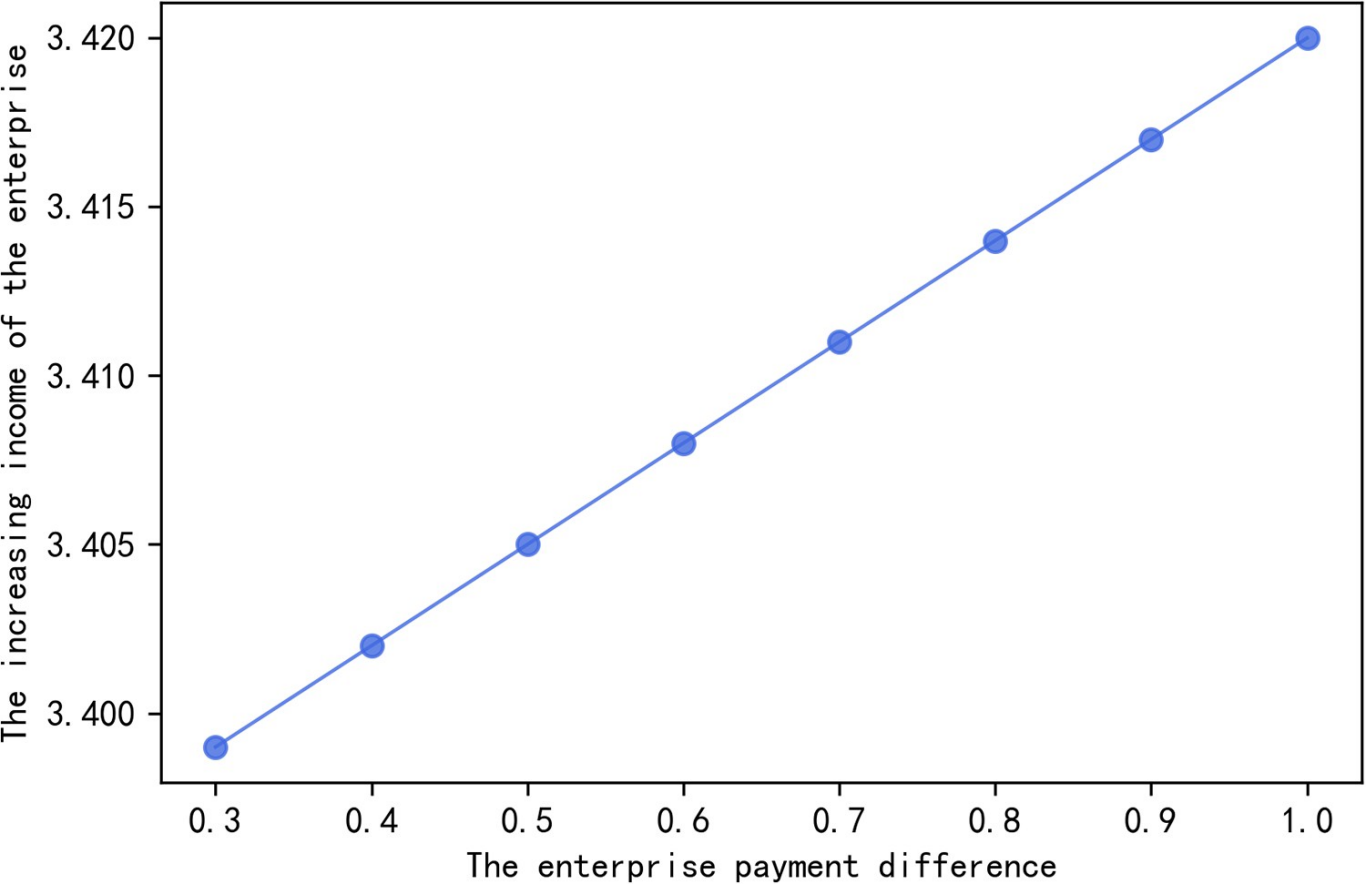
The relationship between enterprise’s added income and EPD.

In order to further explain the impact of enterprise payment difference on different farmers’ effort degree and enterprise’s own income, several groups of data are selected in the effective set for analysis. The calculation results are shown in Table 1, and the changes are shown in Figs 6 and 7.

**Table 1:** With the change of EPD, the change of farmers’ increasing effort degree and enterprises’ increasing income.

Δ*φ*	0.3	0.4	0.5	0.6	0.7	0.8	0.9	1.0
*θ*_1_	0.194	0.192	0.190	0.188	0.186	0.184	0.182	0.180
*θ*_2_	0.297	0.296	0.295	0.294	0.293	0.292	0.291	0.290
Δ*q*_1_	0.0106	0.0208	0.0310	0.0411	0.0540	0.0614	0.0715	0.0817
Δ*q*_2_	0.0002	0.0087	0.0171	0.0255	0.0339	0.0423	0.0506	0.0590
Δ*PZ*	3.399	3.402	3.405	3.408	3.411	3.414	3.417	3.420

Table 1 and Fig 6 illustrate that (i) within the effective set of enterprise payment difference, the effort degree of farmers will increase with the increase of enterprise payment difference; (ii) the increase of effort degree of different farmers is different, and the added value of effort degree is related to the degree of reciprocity preference of farmers. That is to say the higher the degree of reciprocity preference, the more effort input the farmers will be. Furthermore, Table 1 and Fig 7 show that the enterprise income increases with the increase of enterprise payment difference.

## 6. Conclusions

In contract farming, farmers have the private information about its effort on contract fulfillment. Farmers’ low effort is always a key factor restricting the development of contract farming. In this paper, we construct a non-profit index principal-agent model where both enterprises and farmers have the characteristic of reciprocity preference, and provide a suggestion to the enterprise for designing an incentive mechanism to farmers. Our study shows that when farmers have reciprocity preference, enterprises with reciprocity preference can establish an incentive mechanism according to following approaches.

(i) When the enterprise is unable to afford extra rewards to farmers, that is, the EPD is 0, each incentive coefficient should be determined according to the influence of the task on the order income, respectively. The greater the impact is, the greater the incentive coefficient should be. Furthermore, the value of incentive coefficient should be set also based on the farmers’ risk aversion and external risk. Since the payment difference of enterprises has no effect on the efforts of farmers, it is only necessary to set a minimum acceptable value for farmers. Briefly, the enterprise should give strong incentives to the farmers with stable external environment and weak risk aversion who perform important tasks.
(ii) When the enterprise is able to afford extra rewards to farmers, that is, EPD is greater than 0, he can stimulate the farmers’ planting enthusiasm by paying more extra rewards to farmers, providing training in technical education for farmers, supplying free raw materials for farmers and so on. At the same time, for farmers with different degree of reciprocity preference, the enterprise should set up different incentive mechanisms. He should pay promptly attention to the farmers who increase the effort degree firstly and give rewards to them, so as to improve the degree of farmers’ efforts, reduce the default rate of farmers, increase the income of enterprises, realize a virtuous cycle of contract farming market mechanism, and promote the development of contract farming.

This paper analyzes the principal-agent relationship between enterprises and farmers with reciprocity preference. There are many related agents in contract farming; extensive research can be conducted by adding consumers and the government to form a multiple principal-agent relationship between multiple subjects.

## References

[1] Key ND, MacDonald JM. Agricultural contracting: Trading autonomy for risk reduction. Amber Waves. 2006; 4: 26–31.

[2] Niu BZ, Jin DL, Pu XJ. Coordination of channel members’ efforts and utilities in contract farming operations. European Journal of Operational Research. 2016; 255(3): 869–883.

[3] Vicol M, Fold N, Hambloch C, Narayanan S, Helena PN. Twenty-five years of Living Under Contract: Contract farming and agrarian change in the developing world. Journal of Agrarian Change. 2022; 22(1): 3–18.

[4] Wang HH, Wang Y, Delgado MS. The transition to modern agriculture: contract farming in developing economies. American Journal of Agricultural Economics. 2014; 96(5): 1257–1271.

[5] FAO, FAQ: What is contract farming? Contract farming resource centre. Food and Agriculture Organization of the United Nations, Rome, Italy, http://www.fao.org/ag/ags/contract-farming/faq/en/.

[6] Bellemare MF, Lim S. In all shapes and colors: varieties of contract farming. Applied Economic Perspectives and Policy. 2018; 40(3): 379–401.

[7] Ma JJ, Xu XG. Market structure and contract-performance analysis of contract farming. Issues in Agricultural Economy. 2008; 339(3): 301–314.

[8] Liu FQ. Incomplete contract and the barrier to performance: A case research on the farm produce for order. Economic Research Journal. 2003; 4: 22–30+92.

[9] Yang HX, Sun YQ, Ma JJ. A research on the order default for “farmer-supermarket direct-purchase”. Science Research Management. 2019; 248(6): 225–233.

[10] Federgruen A, Lall U, Şimşek AS. Supply chain analysis of contract farming. Manufacturing & Service Operations Management. 2019; 21(2): 361–378.

[11] Pauli ED, Barbieri F, Garcia PS, Madeira TB, Junior V, Scarminio I, et al. Detection of ground roasted coffee adulteration with roasted soybean and wheat. Food Research International. 2014; 61(7): 112–119.

[12] Babich V, Tang SC. Managing opportunistic supplier product adulteration: deferred payments, inspection, and combined mechanisms. Manufacturing & Service Operations Management. 2019; 14(2): 301–314.

[13] Levi R, Singhvi S, Zheng YC. Economically motivated adulteration in farming supply chains. Management Science. 2019; 66(1): 1–18.

[14] Xu P, Wang L, Fu HY, Chen XX. Incentive mechanism between 4PL and 3PL considering reciprocity preference in agricultural product supply chain finance. Management Review. 2019; 31(1): 62–70.

[15] JD.com Cooperates with Northwest Agriculture and Forestry University, Help Yangling Cultivate "New Farmers", sanqin.com, November 27th, 2018, http://www.sanqin.com/2018/1127/395637.shtml.

[16] When E-commerce is Helping Farmers, how does E-commerce for Agricultural Products Go? sohu.com, February 28th, 2020, https://www.sohu.com/a/376456083_115888.

[17] AI Devs Behind Robot Sophia Partner with Blockchain Agricultural Data Firm, cointelegraph.com, January 21, 2019, https://cointelegraph.com/news/ai-devs-behind-robot-sophia-partner-with-blockchain-agricultural-data-firm.

[18] Yang KL, Wu Q. The application of non-profit indicators in performance commitment. Communication of Finance and Accounting. 2019; 835(35): 90–93.

[19] Li B. Analysis of contract incompleteness and default risk of “enterprise + farmer”. Rural Economy. 2009; 318(4): 29–31.

[20] Guo L. Influence of transaction costs, trusting relationship on farmer’s compliance behaviour——Based on survey data from 286 apple growers in Shandong province. Journal of Huazhong Agricultural University (Social Sciences Edition). 2015; 118(4): 56–61.

[21] Wang YF, Huang Y, Tang S. The efficiency and motivation of order fulfilment between leading enterprises and farmers——Survey data from 91 agricultural enterprises, Issues in Agricultural Economy. 2014; 35(11): 16–25.

[22] Liu XY, Zhou L. Agricultural contract arrangement: farmers’ risk attitude and contract choice decision. Journal of Nanjing Agricultural University (Social Sciences Edition). 2020; 92(2): 140–148.

[23] Chen W, Zhang XM, Song H. A study on the relation contract of knowledge trading among members in the supply chain based on the perspective of cooperative innovation. Science Research Management. 2015; 36(7): 38–48.

[24] Ye F, Lin Q, Li Y. Coordination for contract farming supply chain with stochastic yield and demand under CVaR criterion. Operational Research. 2017; 20: 369–397.

[25] Anderson E, Monjardino M. Contract design in agriculture supply chains with random yield. European Journal of Operational Research. 2019; 277(3): 1072–1082.

[26] Cao Y, Tao L, Wu K. Contract design for contract farming under information asymmetry. The Theory and Practice of Finance and Economics. 2021; 232(4): 97–103.

[27] Jiao MC, Xue XL. Game analysis and avoidance strategies of farmers’ default behaviour in contract agriculture. Shandong Social Sciences. 2015; 238(6): 122–126+116.

[28] Loch CH, Wu YZ. Social preferences and supply chain performance: an experimental study. Management Science. 2008; 54(11): 1835–1849.

[29] Ho TH, Su XM, Wu YZ. Distributional and peer-induced fairness in supply chain contract design. Production and Operations Management. 2014; 23(2): 161–175.

[30] Pan KW, Cui ZB, Xing A, Lu QH. Impact of fairness concern on retailer-dominated supply chain. Computers & Industrial Engineering. 2020; 139: 106209.1–106209.9.

[31] Liao YL. Chinese hi-tech enterprise qualification examination and accreditation through the lens of fairness and reciprocity theory. International Journal of Education and Management. 2016; 1(1): 41–47.

[32] Caliskan DO, Chen YH, Li JB. Channel coordination under fairness concerns and nonlinear demand. European Journal of Operational Research. 2010; 207(3): 1321–1326.

[33] Jiang BJ, Ni J, Srinivasan K. Signalling through pricing by service providers with social preferences. Marketing Science. 2014; 33(5): 641–654.

[34] Du SF, Nie TF, Chu CB, Yu YG. Reciprocal supply chain with intention. European Journal of Operational Research. 2014; 239(2): 389–402.

[35] Zhang KY. Analysis on closed-loop supply chain pricing decision under reciprocity preference. Control and Decision. 2015; 30(9):1717–1722.

[36] Ma GW, Meng WD, Dai JS. The principal-agent contract design based on fairness preference and learning effect. Systems Engineering-Theory & Practice. 2017; 37(6):1548–1556.

[37] Xu P. Research on financial incentive contract of online agricultural product supply chain with reciprocal preference. Journal of Commercial Economics. 2019; 14: 165–168.

[38] Ali E. Farmers’ attitudes towards climate risks and effects of farmers’ risk aversion behavior on inputs use in northern togo. Sarhad Journal of Agriculture. 2019; 35(3): 663–674.

[39] Niu BZ, Chen KL, Fang X, et al. Technology specifications and production timing in a co-opetitive supply chain. Production and Operations Management. 2019; 57(10): 1737–751.

[40] Wickens MR, Greenfield JN. The econometrics of agricultural supply: an application to the world coffee market. Review of economics and Statistics. 1973; 55(4): 433–440.

[41] Alizamir S, Iravani F, Mamani H. An analysis of price vs. revenue protection: government subsidies in the agriculture industry. Management Science. 2019; 65(1): 32–49.

[42] Chen CY, Huang BL. A comparative analysis of principal agent model of supply chain financing under reciprocal preference theory. Journal of Business Economics. 2015; 12: 52–60.

[43] Sobel J. Interdependent Preferences and Reciprocity. Journal of Economic Literature. 2005; 43(2): 392–436.

[44] Xu P, Wang Y, He D. Incentive Contract between 4PL and 3PL Based on FWT of unified credit Mode. Journal of Industrial Engineering and Engineering Management. 2012; 26(100): 50–54+108.

[45] Wang Y, Xu P. The incentive mechanism of banks to3PL considering the factor of justice preference based on FTW of principal-agent mode. Journal of industrial Engineering and Engineering Management 2010; 24(01): 95–100.

